# Preparation and Whitening Activity of Sialoglycopeptide of Chalaza from Liquid Egg Process

**DOI:** 10.3390/molecules31010059

**Published:** 2025-12-23

**Authors:** Yanzhao Ma, Ziyi Jiang, Xinyi Jin, Jianrong Wu, Minjie Gao

**Affiliations:** 1 Key Laboratory of Carbohydrate Chemistry and Biotechnology of Ministry of Education, School of Biotechnology, Jiangnan University, Wuxi 214122, China; myz001118@163.com (Y.M.); jmgaoz@jiangnan.edu.cn (M.G.); 2 State Key Laboratory of Food Science and Resource, Jiangnan University, Wuxi 214122, China

**Keywords:** chalaza, sialoglycopeptide, whitening activity, melanin synthesis, tyrosinase activity

## Abstract

The liquid egg processing industry generates a significant amount of solid byproduct known as chalaza (CHA), which is rich in sialic acid and exhibits notable biological activity. In this study, the preparation process, N-glycan profile, and skin-whitening activity of CHA-derived glycopeptides (CHAH) were investigated. By comparing the hydrolysis efficiency of trypsin, alcalase, and papain, a dual-enzyme hydrolysis strategy was developed: initial hydrolysis with 1.5% trypsin for 3 h, followed by treatment with 1% papain for 2 h. The resulting CHAH exhibited both a high hydrolysis yield and strong antioxidant activity. The sialic acid content in CHAH reached 1.96% (*w*/*w*), and 14 distinct N-glycan chain structures were identified. The whitening effect of CHAH was assessed using a combined approach involving an in vitro B16 cell model and an in vivo zebrafish model. CHAH was found to inhibit tyrosinase activity and reduce melanin production in a concentration-dependent manner. Mechanistic studies revealed that CHAH acts by significantly downregulating the expression of key genes involved in melanin synthesis, including MITF, TYR, TYRP1, and TYRP2. This study establishes an efficient preparation method for CHAH, elucidates its skin-whitening efficacy and underlying mechanism, and provides experimental support for the potential industrial application of CHAH as an active ingredient in skincare products.

## 1. Introduction

Melanin is a naturally occurring biological pigment ubiquitously distributed in living organisms. Its primary physiological function is to protect the skin from ultraviolet (UV) radiation-induced damage by absorbing and scattering UV rays, thereby mitigating oxidative damage to skin cell DNA [1]. However, dysregulation of the melanin biosynthetic pathway, which results in melanin overproduction and aberrant deposition, can be a precursor to various pathological conditions. Clinically, this is observed in hyperpigmentation disorders including senile lentigines and melasma, and in malignant melanoma [2]. In the nervous system, the aberrant accumulation of melanin has also been linked to the pathogenesis of neurodegenerative diseases, including Parkinson’s disease (PD). This association may be mediated through mechanisms such as the induction of oxidative stress in neuronal cells [3]. Therefore, targeted regulation of melanogenesis has emerged as one of the key strategies for the clinical management of hyperpigmentation disorders [4]. Studies have demonstrated that the Microphthalmia-associated Transcription Factor (MITF) regulates the expression of the tyrosinase-related protein family, primarily consisting of tyrosinase (TYR), tyrosinase-related protein 1 (TYRP1), and tyrosinase-related protein 2 (TYRP2). These three key enzymes collectively constitute a multi-enzyme complex [5]. In melanoma cells (B16 murine melanoma cell line), TYR is co-expressed with TYRP1 or TYRP2 under the regulation of MITF [6]. Of these, TYRP1 is the key enzyme facilitating the proper trafficking of TYR to melanogenic sites, whereas TYRP2 modulates the catalytic activity of melanogenic proteins in the early stages of melanogenesis [7,8].

Ascorbic acid, kojic acid, hydroquinone, and arbutin are among the recognized regulators of melanogenesis [9]. However, their application is often limited by inherent drawbacks or adverse effects. For example, ascorbic acid is prone to oxidation in whitening formulations, which compromises its efficacy [10]. In contrast, both hydroquinone and arbutin have been linked to genotoxicity and potential carcinogenicity [11,12,13,14]. Notably, numerous studies have confirmed that food-derived bioactive peptides, isolated from a variety of sources, also possess considerable skin-whitening potential [15,16]—including edible bird’s nest [17], pearl oyster meat [18], tuna skin [19,20,21], spirulina [22], soybean [23], and rice [24,25,26]. These peptides demonstrate considerable potential for application across both the food and cosmetic industries, extending from innovative functional food ingredients to active components in topical cosmetic formulations [27].

In China, the annual output of chalaza (CHA)—a principal by-product from liquid egg processing—exceeds 450 metric tons. The prevailing practice for its disposal is through landfilling or incineration, entailing not only substantial resource wastage but also thereby exerting considerable pressure on the environment [28]. However, CHA contains more than 85% protein and is rich in bioactive ovomucin as well as various essential amino acids, making it a highly nutritious resource with considerable potential for development [29]. Previous studies have shown that the crude hydrolysate of CHA (CHAH), obtained by enzymatic hydrolysis with protease A, can mitigate oxidative stress-induced cognitive impairment through multiple pathways, including antioxidant and anti-inflammatory effects, as well as suppression of the AGEs/RAGE pathway [30]. Furthermore, in a thioacetamide (TAA)-induced rat model of hepatic fibrosis, CHAH was found to ameliorate fibrosis via a triple mechanism involving antioxidant, anti-inflammatory, and pro-apoptotic activities [31]. Preliminary analyses have also indicated that CHA contains approximately 2.4% sialic acid, which is likely a key contributor to its diverse bioactivities. Given that CHA hydrolysate may contain sialoglycopeptides with N-glycans similar to edible bird’s nest peptide and exhibits anti-inflammatory and antioxidant effects, we hypothesize that CHA hydrolysates also CHA hydrolysate can also be applied in the field of skincare as a whitening agent.

Based on this background, this study aimed to prepare glycopeptides from chalaza (CHA) and assess their skin-whitening potential. The hydrolysis conditions for CHA were optimized, and the whitening efficacy of the resulting hydrolysate (CHAH) was systematically evaluated using both in vitro B16 cell and in vivo zebrafish embryo models. Furthermore, the regulatory effects of CHAH on the expression of key melanogenesis-related genes were examined. The findings are expected to provide a theoretical basis and experimental evidence to support the further development of CHA in the functional food and cosmetic industries.

## 2. Results

### 2.1. Preparation of CHAH

**Figure 1:**
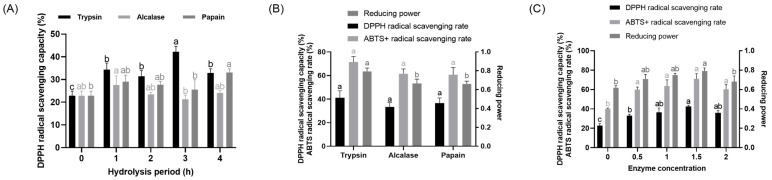
First-stage enzymatic hydrolysis of CHA. Note: (**A**) Hydrolysis time; (**B**) Protease type; (**C**) Protease concentration. DPPH refers to 1,1-Diphenyl-2-picrylhydrazyl; ABTS refers to 2,2′-Azinobis-(3-ethylbenzthiazoline-6-sulfonic acid). L-ascorbic acid (VC) was used as the positive control. Bars with the same letter are not significantly different (*p* > 0.05), while different letters indicate significant differences (*p* < 0.05). Mean values are labeled with “a” for the highest group, and subsequent letters are assigned in descending order based on significant differences. The figure presents the significance analysis conducted for identically colored groups. Each group of parallel samples has a size of *n* = 3.

As single protease treatment generally fails to completely hydrolyze CHA [32], a two-stage enzymatic method was explored. Figure 1 presents the antioxidant activity of CHAH produced under different hydrolysis parameters. Specifically, Figure 1A shows that hydrolysates from all three proteases displayed greater DPPH scavenging capacity than the unhydrolyzed control (0 h group) at an equivalent enzyme activity ratio. The highest activity (*p* < 0.05) was achieved with trypsin after 3 h of hydrolysis. Since prolonging the reaction to 4 h diminished all antioxidant responses, the 3 h duration was identified as the optimum.

As shown in Figure 1B, under a fixed 3 h hydrolysis duration and equivalent enzyme activity, CHAH prepared with trypsin exhibited moderately higher DPPH and ABTS^+^ radical scavenging capacities than the other two proteases, along with significantly greater reducing power (*p* < 0.05). The effect of the trypsin-to-substrate ratio is presented in Figure 1C, which shows that the antioxidant indices of all CHAH samples were significantly elevated compared to the unhydrolyzed CHA control (*p* < 0.05). Among them, the group treated with a 1.5% enzyme dosage demonstrated the highest DPPH scavenging capacity and reducing power (*p* < 0.05). Based on this comprehensive analysis, the optimal conditions for the first hydrolysis stage were determined to be: trypsin, a 3 h duration, and 1.5% trypsin.

**Figure 2:**
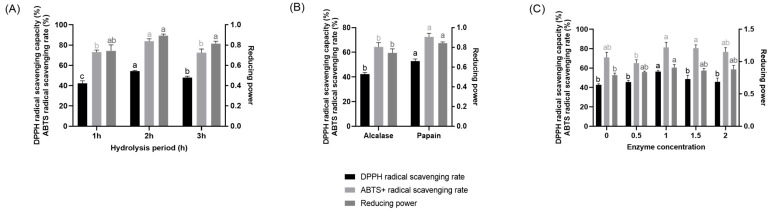
Second-stage enzymatic hydrolysis of CHA. Note: (**A**) Hydrolysis time; (**B**) Protease type; (**C**) Enzyme concentration. DPPH refers to 1,1-Diphenyl-2-picrylhydrazyl; ABTS refers to 2,2′-Azinobis-(3-ethylbenzthiazoline-6-sulfonic acid). L-ascorbic acid (VC) was used as the positive control. Bars with the same letter are not significantly different (*p* > 0.05), while different letters indicate significant differences (*p* < 0.05). Mean values are labeled with “a” for the highest group, and subsequent letters are assigned in descending order based on significant differences. The figure presents the significance analysis conducted for identically colored groups. Each group of parallel samples has a size of *n* = 3.

To further enhance hydrolysis efficiency, a second protease was introduced following the optimized first stage. As shown in Figure 2, CHAH produced by subsequent treatment with 1% papain for 2 h exhibited markedly higher DPPH and ABTS^+^ radical scavenging capacities as well as greater reducing power compared to all other enzymatic conditions tested. On the basis of these results, the optimal second-stage hydrolysis conditions were established as 2 h treatment with 1% papain.

### 2.2. Sialic Acid (N-Acetylneuraminic Acid) Content in CHAH and CHA

**Figure 3:**
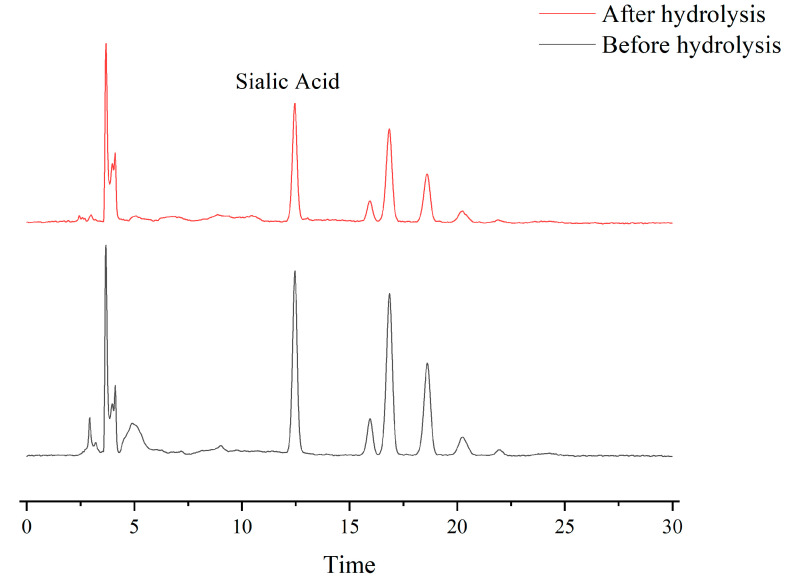
Sialic acid content of CHA before and after hydrolysis. Note: the sample size (*n*) for each group of parallel samples is 3.

CHA is a special biological structure connecting the yolk and albumen in eggs, and its glycopeptides are rich in sialic acid [33]. Sialic acid content was determined before and after hydrolysis, as shown in Figure 3. The results showed that the sialic acid content of unhydrolyzed CHA was 2.29% (*w*/*w*). Notably, CHAH retained a significant sialic acid content of 1.96% (*w*/*w*). Furthermore, approximately 75% of the CHAH peptides had molecular weights below 5 kDa (see Table S1).

### 2.3. N-Glycomic Analysis of CHAH

**Figure 4:**
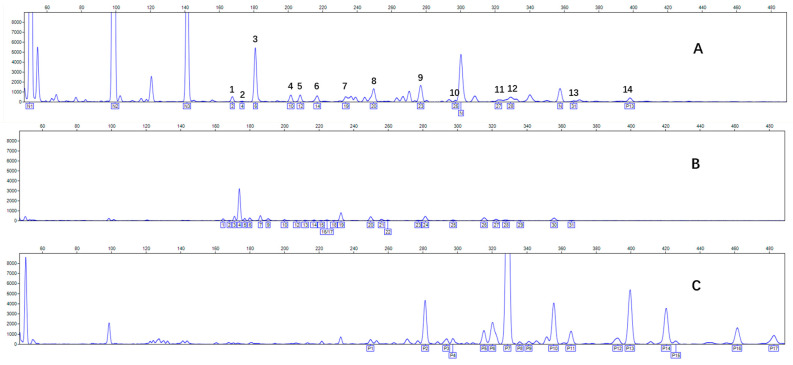
N-glycan profile of CHAH and identified glycan structures [34]. Note: (**A**) CHAH; (**B**) Plasma sample; (**C**) Desialylated plasma sample.

CHA contains diverse glycoproteins bearing sialylated O-glycans and N-glycans. The N-glycan profile of CHAH polypeptides was profiled using DSA-FACE technology, as illustrated in Figure 4. Plasma protein N-glycans were used as the reference standard, and electropherogram peaks were analyzed with dedicated software [34].

A total of 14 distinct N-glycan peaks were detected, several of which have known structures as shown in Table 1. Among them, three peaks (N1, N2, N3) showed differential migration compared to the plasma N-glycan profile and were tentatively assigned as maltose (DP2), maltotriose (DP3), and maltotetraose (DP4) based on their electrophoretic mobility. Most of the fourteen N-glycans identified in CHAH glycoproteins were sialylated, and some were also fucosylated. Excluding N1–N3, the most abundant peak was characterized as the complex-type biantennary glycan A2G2S1(2,6)S1(2,3).

### 2.4. Effect of CHAH on Tyrosinase Activity

**Figure 5:**
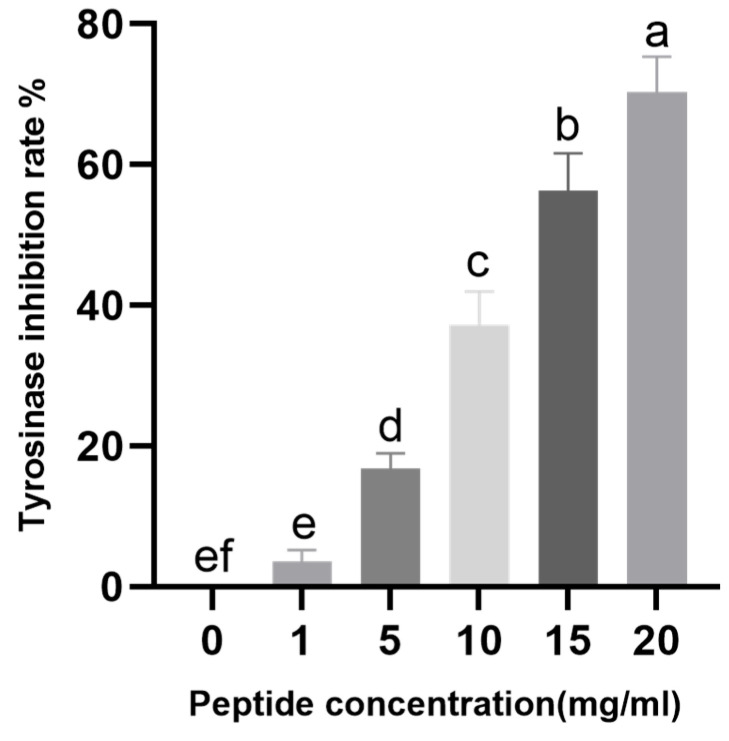
Inhibition of tyrosinase activity in vitro. Note: Bars with the same letter are not significantly different (*p* > 0.05), while different letters indicate significant differences (*p* < 0.05). The group with the highest mean value is labeled “a”, and subsequent groups are assigned letters in descending alphabetical order. The sample size (*n*) for each group of parallel samples is 3.

Tyrosinase, a copper-containing glycoprotein, serves as the key rate-limiting enzyme in the regulation of melanin biosynthesis. The copper ions within its active site interact with molecular oxygen to form highly reactive chemical intermediates, which directly mediate two critical reactions catalyzed by tyrosinase: the hydroxylation of monophenols to o-diphenols, followed by oxidation of o-diphenols to o-quinones [36]. As shown in Figure 5, CHAH exhibited a dose-dependent inhibitory effect on tyrosinase activity within the concentration range of 1–20 mg/mL, with inhibition rates increasing from 3.56% to 70.38%. These results demonstrate that CHAH possesses a potent capacity to inhibit tyrosinase activity. Furthermore, statistical analysis indicates that at a concentration of 13.80 mg/mL, CHAH achieves a 50% inhibition rate against tyrosinase, corresponding to an IC_50_ value of 13.30 mg/mL. Table 2 presents the comparative IC50 values for tyrosinase inhibition. Notably, the IC50 of CHAH is approximately twice that of the EBN glycopeptides, a difference that may be attributed to the lower sialic acid content in CHAH.

### 2.5. Analysis of the In Vitro Skin-Whitening Efficacy of CHAH

**Figure 6:**
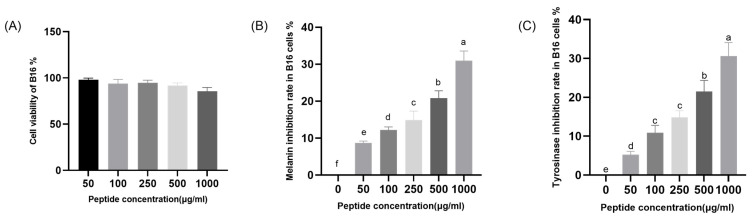
Effects of CHAH on cell viability (**A**), melanin inhibition rate (**B**), and tyrosinase activity (**C**) in B16 cells. Note: Arbutin (1000 μg/mL) was served as the positive control. Bars with the same lowercase letter are not significantly different (*p* > 0.05), while different letters indicate significant differences (*p* < 0.05). The group with the highest mean value is labeled “a”, and subsequent groups are assigned letters in descending alphabetical order. The sample size (*n*) for each group of parallel samples is 3.

Safety assessment is an essential prerequisite for the research and application of bioactive substances. In this study, the skin-whitening effect of CHAH was evaluated using B16 melanoma cells, as summarized in Figure 6. Within the concentration range of 50–1000 μg/mL, CHAH did not significantly inhibit the proliferation of B16 cells or induce noticeable alterations in cell morphology (Figure 6A). As the concentration of CHAH increased from 50 μg/mL to 1000 μg/mL, its inhibitory effect on melanin synthesis in B16 cells rose progressively from 8.68% to 30.98% (Figure 6B). These results demonstrate that CHAH significantly suppresses melanin production in B16 cells in a concentration-dependent manner (*p* < 0.05). Furthermore, the inhibitory effect of CHAH on intracellular tyrosinase activity was assessed. As shown in Figure 6C, over the concentration range of 50–1000 μg/mL, the tyrosinase inhibition rate increased steadily from 5.20% to 30.61%, also exhibiting a significant concentration-dependent relationship. This finding suggests that CHAH effectively suppresses intracellular tyrosinase activity in B16 cells. Collectively, these data indicate that CHAH possesses notable skin-whitening activity in the B16 cell in vitro model. This section clarifies the rationale behind the experimental design to address potential concerns. The selection of 1000 μg/mL (1 mg/mL) as the maximum test concentration was based on two key factors, both validated in preliminary experiments: cellular safety and practical applicability. First, cell viability assays (Figure 6A) showed that when CHAH concentrations exceeded 1000 μg/mL (e.g., 1500 μg/mL or 2000 μg/mL), B16 cell viability dropped below 80% (*p* < 0.05). This reduction in cell survival introduces interference from cell death in melanin and tyrosinase assays, which violates the requirement for a “non-cytotoxic concentration range” necessary to evaluate melanogenesis-related activity accurately [7,37]. Second, solubility tests confirmed that 1000 μg/mL is the saturation concentration of CHAH in DMEM medium (supplemented with 10% fetal bovine serum). Higher concentrations led to visible precipitation, causing uneven sample distribution in 96-well plates and inaccurate absorbance readings—a common technical challenge in studies involving natural peptide extracts [37]. Additionally, this concentration aligns with the typical application doses for natural active ingredients in cosmetic formulations, ensuring the practical relevance of the results [7,20].

### 2.6. In Vivo Skin-Whitening Efficacy of CHAH in Zebrafish Embryos

**Figure 7:**
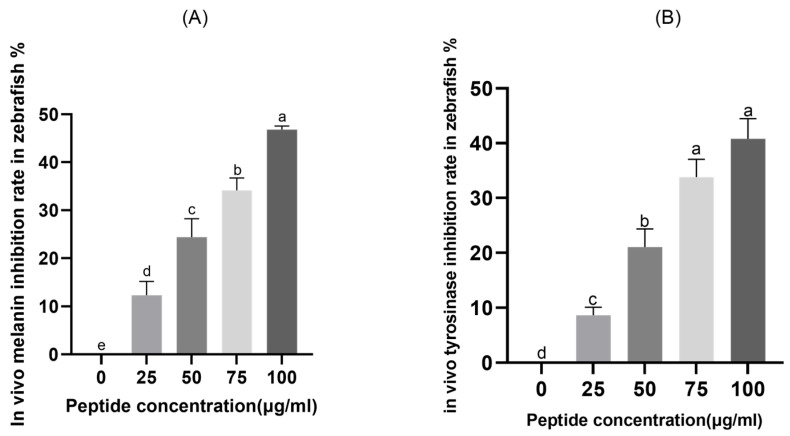
Effects of CHAH on melanin inhibition rate (**A**) and tyrosinase activity (**B**) in zebrafish. Note: Arbutin (100 μg/mL) was served as the positive control. Bars with the same lowercase letter are not significantly different (*p* > 0.05), while different letters indicate significant differences (*p* < 0.05). The group with the highest mean value is labeled “a”, and subsequent groups are assigned letters in descending alphabetical order. The sample size (*n*) for each group of parallel samples is 3.

Zebrafish embryos represent a valuable vertebrate model for evaluating skin-whitening agents, owing to their optical transparency during early developmental stages and the initiation of melanin synthesis in the retinal epithelium around 24 h post-fertilization. In this work, zebrafish embryos were treated with various concentrations of CHAH (25, 50, 75, and 100 μg/mL). As illustrated in Figure 7A, CHAH significantly inhibited melanin synthesis in a concentration-dependent manner, with inhibition rates of 12.32%, 24.41%, 34.15%, and 46.80%, respectively. The inhibitory effect of CHAH on tyrosinase activity in zebrafish embryos was also assessed (Figure 7B). With increasing CHAH concentrations from 25 to 100 μg/mL, tyrosinase inhibition rose progressively from 8.66% to 40.80%. All tested concentrations resulted in statistically significant differences compared to the control group (*p* < 0.05), further supporting the concentration-dependent inhibitory activity of CHAH.

### 2.7. Skin-Whitening Mechanism of CHAH

#### 2.7.1. CHAH Downregulates Melanogenesis-Related Genes Expression in B16 Cells

Melanogenesis is tightly regulated by a cascade of key genes, each of which plays an essential role in the biosynthetic pathway. Microphthalmia-associated Transcription Factor (MITF) acts as the master regulator of melanocyte development and melanin synthesis. It directly binds to the promoters of tyrosinase family genes to initiate their transcription [38,39]. Tyrosinase (TYR) is the rate-limiting enzyme that catalyzes the initial and rate-determining steps in melanin production: the hydroxylation of L-tyrosine to L-DOPA, followed by its oxidation to dopaquinone [6,36]. Tyrosinase-related protein 1 (TYRP1) is crucial for the proper trafficking of TYR to melanosomes and the stabilization of melanogenic complexes. Meanwhile, TYRP2 (dopachrome tautomerase) regulates the conversion of dopachrome to 5,6-dihydroxyindole-2-carboxylic acid (DHICA) during the early stages of eumelanin synthesis [6,7].

Given the critical and non-redundant roles of these four genes in melanin biosynthesis, measuring their expression levels provides a direct and reliable way to explore the molecular mechanisms underlying the anti-melanogenic effects of CHAH. This approach aligns with widely accepted research paradigms in skin-whitening studies [7,37], ensuring the validity and comparability of our mechanistic findings.

**Figure 8:**
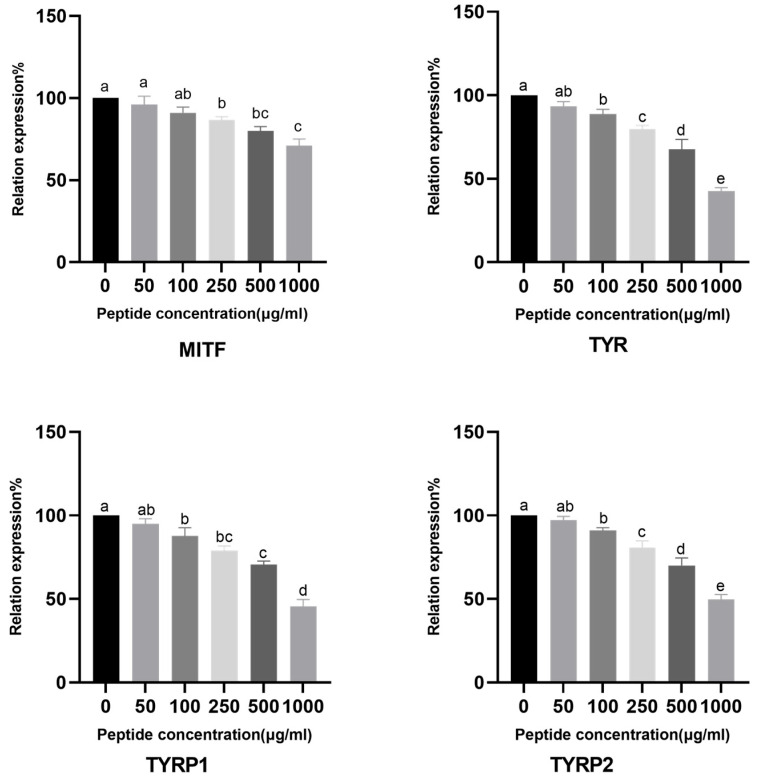
Effect of CHAH on the expression of MITF, TYR, TYRP1, and TYRP2 genes in B16 cells. Note: MITF refers to Microphthalmia-associated Transcription Factor; TYR refers to Tyrosinase; TYRP1 refers to Tyrosinase-related protein 1; TYRP2 refers to Tyrosinase-related protein 2. Arbutin (1000 μg/mL) was served as the positive control. Bars with the same lowercase letter are not significantly different (*p* > 0.05), while different letters indicate significant differences (*p* < 0.05). The group with the highest mean value is labeled “a”, and subsequent groups are assigned letters in descending alphabetical order. The sample size (*n*) for each group of parallel samples is 3.

To understand how CHAH regulates melanin synthesis at the molecular level, we investigated its effect on the expression of these four key genes—MITF, TYR, TYRP1, and TYRP2—in B16 cells. As shown in Figure 8, CHAH treatment resulted in a concentration-dependent suppression of all four genes. At the highest concentration tested (1000 μg/mL), CHAH significantly downregulated the expression of MITF, TYR, TYRP1, and TYRP2 by 29.00%, 57.33%, 54.33%, and 50.46%, respectively, compared to the control group.

#### 2.7.2. CHAH Downregulates Melanogenesis-Related Genes Expression in Zebrafish

**Figure 9:**
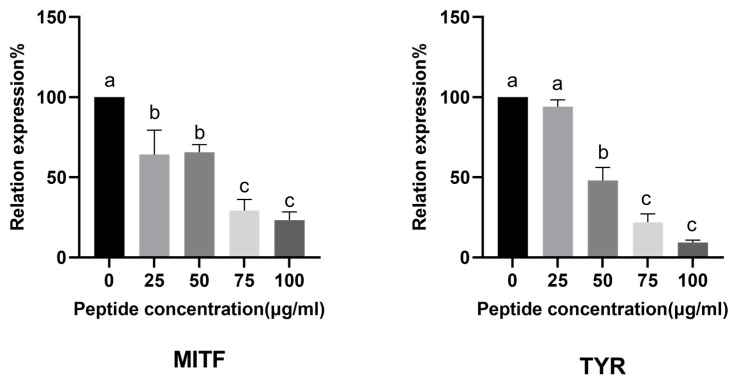
Effect of CHAH on the expression of MITF and TYR genes in zebrafish. Note: MITF refers to Microphthalmia-associated Transcription Factor; TYR refers to Tyrosinase; TYRP1 refers to Tyrosinase-related protein 1; TYRP2 refers to Tyrosinase-related protein 2. Arbutin (1000 μg/mL) was served as the positive control. Bars with the same lowercase letter are not significantly different (*p* > 0.05), while different letters indicate significant differences (*p* < 0.05). The group with the highest mean value is labeled “a”, and subsequent groups are assigned letters in descending alphabetical order. The sample size (*n*) for each group of parallel samples is 3.

Having established that CHAH significantly suppresses melanogenesis and tyrosinase activity, we further investigated its in vivo mechanism by evaluating the expression of key melanogenesis-related genes in zebrafish. As shown in Figure 9, CHAH treatment led to a concentration-dependent downregulation of MITF and TYR expression. Compared with the control group, CHAH at concentrations of 25–100 μg/mL significantly reduced MITF expression (*p* < 0.05), while TYR expression was significantly suppressed at 50–100 μg/mL (*p* < 0.05). The highest concentration of CHAH (100 μg/mL) strongly downregulated MITF and TYR expression by 76.33% and 90.33%, respectively. These results demonstrate that CHAH inhibits melanogenesis in zebrafish by suppressing the expression of MITF and TYR.

## 3. Discussion

This work establishes a two-stage enzymatic hydrolysis process for the high-value conversion of egg-processing by-product chalaza (CHA) into glycopeptides (CHAH). The resulting CHAH contained 75% of peptides with molecular weights below 5 kDa, which aligns with the reported enhanced bioavailability of naturally derived skin-whitening peptides from sources such as edible bird’s nest and pearl oyster meat [7,18]. N-glycomic profiling further identified 14 distinct N-glycan structures in CHAH, most of which were sialylated or fucosylated—a finding consistent with the inherent glycoprotein-rich nature of CHA [29]. Previous evidence indicates that sialic acid can enhance bioactivity by modulating cellular signaling pathways [33], while fucosylation often improves glycopeptide stability and targeting specificity. The synergy between these glycosylation modifications may thus represent a key molecular basis for the skin-whitening activity observed in CHAH.

Notably, the skin-whitening activity of CHAH appears intrinsically linked to its potent antioxidant capacity [40]. This connection merits further exploration through the regulatory axis involving reactive oxygen species (ROS), inflammatory factors, and melanin synthesis, as well as the potential role of ovomucin—a key bioactive component in CHA. The relationship between antioxidant activity and skin whitening is well established: under external stimuli such as UV radiation, skin accumulates substantial ROS [41], which in turn enhance the activity of MITF, the central transcription factor in melanogenesis, via activation of the RAS/ERK signaling pathway [42]. This leads to upregulated expression and activation of key enzymes including TYR and TYRP1, ultimately promoting melanin production and deposition [40]. In the present study, CHAH prepared by two-stage enzymatic hydrolysis demonstrated strong antioxidant performance, with its DPPH and ABTS^+^ radical scavenging rates and reducing power significantly surpassing those of unhydrolyzed CHA (Figure 1 and Figure 2). This robust antioxidant capacity likely enables CHAH to scavenge ROS at the source, thereby disrupting the “oxidative stress–melanogenesis activation” cascade and providing a mechanistic foundation for its observed skin-whitening efficacy.

Furthermore, the synergistic mechanism of melanogenesis involving ROS and inflammatory factors reveals that excessive ROS not only directly promotes melanin synthesis [43] but also triggers the secretion of pro-inflammatory cytokines such as TNF-α and IL-1β from keratinocytes and immune cells. These cytokines bind to receptors on melanocytes, enhancing the nuclear localization and stability of MITF, which in turn leads to sustained upregulation of TYR transcription and enzymatic activity [44]. Together, these processes form a self-reinforcing vicious cycle of “ROS accumulation–inflammatory activation–hyperpigmentation.”

The ability of CHA to disrupt this cycle can be largely attributed to the biological properties of ovomucin, a major protein constituent of CHA. With a protein content exceeding 85%, CHA is notably rich in ovomucin, which possesses both antioxidant and anti-inflammatory functions. Previous studies have indicated that CHA hydrolysates containing bioactive ovomucin fragments can elevate the activities of superoxide dismutase (SOD) and glutathione peroxidase (GSH-Px), thereby reducing ROS levels [45], while simultaneously downregulating the expression of inflammatory cytokines including TNF-α and IL-1β to alleviate associated oxidative and inflammatory damage [46]. Through this dual antioxidant and anti-inflammatory mode of action, CHAH synergistically suppresses the melanogenic pathway, thereby providing critical mechanistic support for its observed skin-whitening efficacy [44].

Both in vitro (B16 cells) and in vivo (zebrafish) models confirmed CHAH’s skin-whitening activity. No significant cytotoxicity or adverse reactions were observed within the tested concentration range, highlighting its excellent safety profile. This distinguishes CHAH from many traditional chemical whitening agents, with its safety characteristics aligning with bioactive peptides from natural sources such as tuna skin and spirulina [22]. Molecular studies indicate that CHAH regulates melanogenesis by downregulating MITF, TYR, TYRP1, and TYRP2 gene expression. Reduced MITF expression inhibits transcription of downstream genes in the tyrosinase family [38,39]. These findings align with other natural bioactive peptide studies. For instance, Bai et al. [7] confirmed that edible bird’s nest peptides (1000 μg/mL) inhibit approximately 35% of melanin production, while Li et al. [37] observed concentration-dependent anti-melanogenesis effects in marine peptides, exhibiting an activity pattern similar to CHAH within the 50–1000 μg/mL concentration range. This confirms CHAH’s efficacy and aligns with the established concentration range for natural whitening peptides (Figure 6B,C). Mechanistically, Kose and Oncel [22] demonstrated that spirulina peptides inhibit pigmentation by downregulating MITF/TYR, while CHAH similarly targets MITF, TYR, TYRP1, and TYRP2 (Figure 8), supporting its whitening potential.

MAPK (including the ERK, p38, and JNK subfamilies) regulates MITF expression and activity in a manner analogous to the PI3K/Akt signaling pathway [38,44]. Activation of the ERK pathway promotes MITF degradation, whereas activation of the PI3K/Akt pathway maintains MITF stability. These two pathways indirectly influence the expression of downstream genes (such as TYR and TYRP1) by regulating MITF [44,45]. Our work confirms that CHAH downregulates MITF and related gene expression in a concentration-dependent manner. Given the similarity in signaling pathway regulation patterns observed with natural whitening peptides (e.g., tuna skin peptide and spirulina peptide) [22,37], it is hypothesized that CHAH may exert its whitening effect by activating the ERK pathway or inhibiting the PI3K/Akt pathway, thereby reducing MITF activity or stability. This hypothesis requires further validation through Western blot analysis of phosphorylation levels of key pathway proteins such as p-ERK and p-PI3K. Subsequent experiments will focus on deepening the investigation of its mechanism of action.

CHAH demonstrates a favorable safety profile for applications in both cosmetics and functional foods. In vitro, B16 cell viability remained >80% at concentrations up to 1000 μg/mL (Figure 6A). zebrafish embryos treated with 100 μg/mL showed no mortality or deformities at 72 hpf, consistent with the established safety profile of egg-derived ingredients [29]. Subsequent key testing consists of a human patch test for cosmetic skin sensitization and a 90-day oral toxicity study for functional foods [14]. Both are feasible and essential for regulatory compliance and meeting industry safety standards.

This study has several limitations. The bioactivity assessment of CHAH was conducted using crude extracts, and the specific contributions of individual peptides or sugar structures to skin whitening effects remain to be isolated and identified. Future research should employ chromatographic separation, mass spectrometry analysis, and molecular docking techniques to precisely identify core active components. Furthermore, while zebrafish models have validated its in vivo efficacy, further validation in mammalian systems more closely resembling human skin physiology—such as mouse skin pigmentation models—would enhance the reliability of these findings. To further ensure regulatory compliance and strengthen the study’s conclusions, skin irritation (OECD 404) and subacute oral toxicity (OECD 408) tests should also be conducted. Such data would provide more direct evidence for the safety and efficacy of CHAH in industrial applications.

From an industrial perspective, this study not only introduces a potential novel natural whitening ingredient but also establishes a technical pathway for the valorization of agricultural by-products [28]. It demonstrates a feasible approach to converting these by-products into high-value active ingredients, thereby enhancing their economic potential. Subsequent research should focus on critical application-oriented properties of CHAH, such as its thermal stability during food processing, storage stability in cosmetic formulations, and compatibility with various delivery systems (e.g., oral liquids and mask matrices), to facilitate its transition from laboratory research to commercially viable products.

## 4. Materials and Methods

### 4.1. Materials

The egg yolk membrane was provided by Jiangsu Tiancheng Egg Industry Co., Ltd. (Nantong, China). Trypsin, alkaline protease, and papain were purchased from Gaoya Biotechnology (Shanghai, China), Sail Biotechnology (Beijing, China), and Sinopharm Chemical Reagents (Shanghai, China), respectively. Tyrosinase and L-tyrosine were purchased from McLean Biochemical (Shanghai, China). Cell culture reagents included Dulbecco’s Modified Eagle Medium (DMEM) with high glucose, supplied by Chemical Reagents Co., Ltd. (Shanghai, China). Tyrosinase and L-tyrosine were purchased from McLean Biochemical (Shanghai, China). Cell culture reagents comprised high-glucose Dulbecco’s Modified Eagle Medium (DMEM) and fetal bovine serum (FBS), both purchased from HyClone (Logan, UT, USA). Mouse melanoma (B16) cells were purchased from Lanxess Biotechnology (Wuxi, China). Molecular biology reagents included the TRIzol™ Kit and Total RNA Extraction Kit, both purchased from TransGen Biotechnology (Beijing, China); the reverse transcription kit was purchased from Bristol-Myers Squibb Biotechnology (Shanghai, China). All other chemical reagents were of analytical grade and supplied by Sinopharm Chemical Reagents (Shanghai, China).

### 4.2. Preparation of CHAH

The preparation method for CHAH followed the previously described procedure [47] with minor modifications. The specific steps are as follows: First, thoroughly rinse the CHA three times with ultrapure water. Subsequently, perform dialysis using a dialysis bag with a molecular weight cut-off (MWCO) of 500 Da, employing ultrapure water as the dialysate. After dialysis, freeze-dry the sample and store it at −20 °C for subsequent use. Next, the CHA sample was mixed with ultrapure water at a preset mass ratio (substrate: water = 1: 20, *w*/*v*) to form a substrate solution, which was hydrolyzed with papain, alcalase, and trypsin separately. The optimal protease and primary hydrolysis conditions were established by systematically comparing the effects of different hydrolysis durations and enzyme concentrations on the degree of hydrolysis (DH)—a key indicator of hydrolysis efficiency. The enzymatic reaction was terminated by heating the mixture in a 95 °C water bath for 10 min. Following the first hydrolysis under optimized conditions and enzyme inactivation, a second protease was added to the system to conduct further hydrolysis. At this stage, the DPPH radical scavenging rate was used as an activity indicator to further optimize the secondary hydrolysis parameters. Finally, the hydrolysate was subjected to a second enzyme inactivation step in a 95 °C water bath, followed by centrifugation at 8000 r/min (4 °C) for 15 min. The resulting supernatant was collected and freeze-dried to obtain the final CHAH product, which was sealed and stored at −20 °C until use.

### 4.3. Antioxidant Activity Assays

The DPPH radical scavenging activity and ABTS radical scavenging activity were assayed according to the method of Ak & Gülçin [48], while the reducing power was determined following the protocol of Yang et al. [47]. Ascorbic acid was used as the positive control.

### 4.4. Determination of Sialic Acid Content

The sialic acid content in CHAH was quantified via High-Performance Liquid Chromatography (HPLC) method [49,50] with minor modifications. Briefly, an accurately weighed amount (approximately 50 mg) of CHAH sample was hydrolyzed in 4 mol/L acetic acid at 80 °C for 3 h in a water bath to ensure complete release of sialic acid. After cooling to room temperature, the released sialic acid was derivatized with o-phenylenediamine (OPD) by incubating in the dark at 80 °C for 40 min using a metal bath. Prior to HPLC analysis, centrifuge the derivatized samples at 4 °C and 12,000 rpm for 10 min, then filter the supernatant through a 0.22-micron membrane filter. Chromatographic separation was performed using an Agilent 1260 HPLC system (Santa Clara, CA, USA) equipped with a Neo-Novi Taurus C18-HP 100A column (4.6 mm × 250 mm, 5 μm) (Neo-Novi (Guangzhou) Technology Co., Ltd., Guangzhou, China). Fluorescence detection was set at an excitation wavelength of 373 nm and an emission wavelength of 448 nm. The mobile phase consisted of a mixture of ultrapure water, acetonitrile, and methanol (volume ratio 85:8:7). The flow rate was maintained at 0.9 mL/min, with an injection volume of 10 μL. A standard curve was constructed using N-acetylneuraminic acid (Neu5Ac) to quantitatively determine neuraminic acid content, expressed as mg/g CHAH.

### 4.5. N-Glycan Profiling of CHAH

The N-glycan structures of CHAH were characterized using DNA sequencer-assisted fluorophore-assisted carbohydrate electrophoresis (DSA-FACE), performed on a Glycome 316 Glyco-Analyzer (Nanjing Suyuan Genomics Technology, Nanjing, China) [34].

### 4.6. Inhibitory Effect of CHAH on Tyrosinase Activity

Inhibitory effect of CHAH with different concentrations on tyrosinase activity was detected according to the protocol described by Li et al. [37] with slight modifications. Briefly, 50 mM phosphate-buffered saline (PBS, pH 6.8) (40 μL), solutions of CHAH (prepared from lyophilized CHAH with different concentrations of 1, 5, 10, 15 and 20 mg/mL) (40 μL) and tyrosinase (236 U/mL) (40 μL) were added sequentially into a 96-well microplate. After thorough vortexing to make the mixture well dispersed, L-tyrosine (1.5 mg/mL) (20 μL) was added carefully into above reaction system to start the enzymatic reaction. Reaction system was incubated at 28 °C for 10 min and then the absorbance was measured at the maximum absorption wavelength of 475 nm by using a microplate reader (Multiskan Sky, Thermo Fisher Scientific, Waltham, MA, USA). Tyrosinase inhibition rate (%) was calculated by the following Equation (1):

(1) $$Tyrosinase\;Inhibition\;Rate\;(\%)\;=\;[1\;-\;(A_{4}\;-\;A_{3})/(A_{2}\;-\;A_{1})]\;\times \;100,\;$$

where A_1_ denotes the absorbance of the system containing only PBS (pH 6.8) and tyrosinase, in the absence of both the sample and substrate; A_2_ denotes the absorbance of the complete reaction system (PBS, tyrosinase, and L-tyrosine) without the sample; A_3_ denotes the absorbance of the system containing PBS, the sample, and tyrosinase, in the absence of the substrate; and A_4_ denotes the absorbance of the complete reaction system containing all components (PBS, sample, tyrosinase, and L-tyrosine).

### 4.7. Effect of CHAH on Melanogenesis in B16 Cells

#### 4.7.1. Cell Culture of B16 Melanoma Cells

B16 melanoma cells in the logarithmic growth phase were collected and adjusted to a final density of 5 × 10^5^ cells/mL. The cell suspension was seeded into 6-well plates at a volume of 2 mL per well. The plates were incubated for 24 h in a humidified atmosphere containing 5% CO_2_ in air at 37 °C to allow for complete cell adhesion. After incubation, the original medium was carefully aspirated and replaced with fresh medium supplemented with different concentrations of lyophilized CHAH. The cells were further cultured for 6 days, and the drug-containing medium was replaced every 48 h with fresh medium of the same concentration. At the end of the culture period, the medium was discarded, and the cells were gently rinsed twice with pre-chilled phosphate-buffered saline (PBS, pH 7.4). Each well was then treated with 0.25% (*v*/*v*) trypsin-EDTA solution to induce cell detachment. Following complete dissociation, the cell suspension was collected into centrifuge tubes and centrifuged at 1000× *g* for 5 min at 4 °C. The supernatant was discarded to obtain the B16 cell pellet. Cell viability was assessed using the Cell Counting Kit-8 (CCK-8, Dojindo Laboratories, Kumamoto, Japan) assay [7].

#### 4.7.2. Determination of Melanin Content in B16 Melanoma Cells

The intracellular melanin content was determined following the previous protocol [7] with minor modifications. Briefly, harvested B16 melanoma cell pellets were resuspended in 200 μL of 1.0 mol/L NaOH solution containing 10% (*v*/*v*) dimethyl sulfoxide (DMSO). The mixture was vortexed vigorously and incubated in an 80 °C water bath for 1 h to ensure complete solubilization of melanin. Following solubilization, 150 μL of the melanin extract was transferred to triplicate wells of a 96-well microplate. The absorbance was measured at 405 nm using a microplate reader. The melanin inhibition rate was calculated using the following formula:

(2) $$Melanin\;Inhibition\;Rate\;(\%)\;=\;[(A_{9}\;-\;A_{10})/(A_{9}\;-\;A_{8})]\;\times \;100,$$

where A_8_ denotes the absorbance of the blank control (melanin extraction solution only, no cells or CHAH); A_9_ denotes the absorbance of the negative control group (B16 melanoma cells + melanin extraction solution, no CHAH); and A_10_ denotes the absorbance of the experimental group (B16 melanoma cells + melanin extraction solution + CHAH). All measurements were performed in triplicate to ensure data reproducibility.

#### 4.7.3. Inhibition of Intracellular Tyrosinase Activity in B16 Melanoma Cells

Intracellular tyrosinase activity inhibition assay was performed according to the procedure described by Bai et al. [7] with slight modifications. After the end of above incubation, B16 melanoma cells were washed twice with phosphate-buffered saline (PBS, pH 7.4). Then, 100 μL of 1% (*v*/*v*) of Triton X-100 were added into each well and the plates were incubated at −80 °C for 60 min at reverse way to promote cell lysis. After cell lysis, 20 μL of cell lysate were added into the corresponding wells individually, 10 μL of L-tyrosine solution (1 mg/mL) was further added, and the mixture was incubated at 37 °C for 1 h. Finally, the absorbance of each well was measured at 450 nm by microplate reader. The tyrosinase inhibition rate was calculated by Formula (1). The parameters represent the corresponding reaction components in this cellular assay system.

#### 4.7.4. Expression of Melanogenesis-Related Genes in B16 Melanoma Cells

Harvested B16 melanoma cells were lysed with 1 mL of TRIzol reagent, vigorously homogenized, and transferred to an RNase-free microcentrifuge tube, followed by vortexing vigorously for 5 min. Subsequently, 200 μL of chloroform was added, the tube was sealed tightly and vortexed vigorously until the solution turned milky white, and then incubated at room temperature for 5 min. The mixture was centrifuged at 12,000× *g* (4 °C) for 15 min. The upper aqueous phase was carefully transferred to a new RNase-free 1.5 mL microcentrifuge tube, avoiding contact with the intermediate protein layer. An equal volume of isopropanol was added to the collected supernatant, gently inverted to mix thoroughly, and incubated at room temperature for 10 min. The sample was centrifuged again at 12,000× *g* (4 °C) for 10 min, resulting in an RNA pellet at the bottom of the tube. The supernatant was discarded, and the pellet was washed with 1 mL of 70% (*v*/*v*) ethanol via gentle inversion, followed by centrifugation under the same conditions for 5 min. After discarding the ethanol, the pellet was air-dried at room temperature for 2–5 min and resuspended in an appropriate volume of RNase-free water (e.g., 20 μL) via gentle pipetting. The dissolved RNA was incubated on ice for ~30 min to ensure complete dissolution. Relative mRNA expression levels were determined by reverse transcription-polymerase chain reaction (RT-PCR) using a commercial kit (TransGen Biotech, Beijing, China). Primers specific to the housekeeping gene (β-actin) and target genes (TYR, TYRP1, TYRP2, and MITF) are listed in Table 3. Relative quantification of TYR, TYRP1, TYRP2, and MITF gene expression was performed using the 2^−ΔΔCt^ method.

### 4.8. In Vivo Skin-Whitening Activity of CHAH

#### 4.8.1. Zebrafish Rearing and Survival Assay

Wild-type zebrafish were obtained from the China Zebrafish Resource Center (Wuhan, China) and maintained in a zebrafish rearing system (Haisheng Biotechnology, Shanghai, China) under controlled conditions of 28.0 ± 1.0 °C and pH 8.0 ± 0.5. The fish were maintained under a 14 h light/dark cycle and fed three times daily. For embryo collection, male and female fish were placed in spawning tanks overnight, and dividers were removed the next morning to allow natural spawning. Collected embryos were cultured in embryo culture medium (ECM) at 28.0 ± 1.0 °C for subsequent in vivo experiments [7,37]. Animal care and procedures strictly adhere to the Regulations on the Care and Use of Laboratory Animals and were approved by the Animal Ethics Committee of Jiangnan University (Jiangsu, China) (Approval No.: JN.No 20240615c0660930).

Healthy zebrafish embryos at 0.75 h post-fertilization (hpf) were selected and transferred into 6-well plates (30 embryos per well), each well containing 3 mL of ECM supplemented with CHAH at final concentrations of 50–750 μg/mL. The plates were incubated in the dark at 28.0 ± 1.0 °C. Embryo viability was monitored and documented using a stereomicroscope (S9i Model, Leica, Wetzlar, Germany) up to 72 hpf.

#### 4.8.2. Determination of Melanin Content and Tyrosinase Activity in Zebrafish

Healthy zebrafish embryos at 6–8 h post-fertilization (hpf) were placed in 6-well plates (30 embryos per well) containing 3 mL of CHAH solution (50–250 μg/mL) prepared in embryo culture medium (ECM), followed by incubation in the dark at 28.0 ± 1.0 °C. Following development to 48 hpf, the embryos were washed twice with ECM and homogenized in 150 μL of 5.0 mg/mL sodium deoxycholate via ultrasonication (VCX130, Sonics, Newtown, CT, USA) at 4 °C. The homogenate was centrifuged at 10,000× *g* (4 °C) for 5 min. The pellet was used for melanin quantification, and the supernatant was collected for tyrosinase activity assay.

Melanin Quantification: The pellet was dissolved in 150 μL of 1.0 mol/L NaOH, and the absorbance was measured at 405 nm. Arbutin (100 μg/mL) was served as the positive control, and the melanin inhibition rate was calculated using Formula (2).

Tyrosinase activity assay: 100 μL of the supernatant was mixed with 100 μL of 5.0 mmol/L L-DOPA solution and incubated in the dark at 37 °C for 1 h. The absorbance was measured at 475 nm, and the tyrosinase inhibitory activity was calculated using Formula (1).

#### 4.8.3. Expression of Melanogenesis-Related Genes in Zebrafish

Zebrafish tissues were minced and homogenized in 1 mL of TRIzol reagent, transferred to an RNase-free microcentrifuge tube, and vortexed vigorously for 5 min. Subsequently, 200 μL of chloroform was added, and the mixture was shaken vigorously until emulsified and milky white, followed by incubation at room temperature for 5 min. The sample was centrifuged at 12,000× *g* (4 °C) for 15 min. After stratification into a colorless upper aqueous phase, a white protein interphase, and a colored lower organic phase, the upper aqueous phase was carefully transferred to a new 1.5 mL microcentrifuge tube, avoiding contamination from the intermediate layer. An equal volume of isopropanol was added to the supernatant, mixed by inversion, and incubated at room temperature for 10 min. The sample was centrifuged at 12,000× *g* (4 °C) for 10 min, yielding a visible pellet. The supernatant was discarded, and the pellet was washed with 1 mL of 70% ethanol by gentle inversion until detached, followed by centrifugation under the same conditions for 5 min. After discarding the ethanol, the pellet was air-dried at room temperature for 2–5 min and resuspended in 20 μL of RNase-free water by gentle pipetting. The RNA was dissolved by incubation on ice for approximately 30 min. Reverse transcription was performed according to the manufacturer’s instructions of the Reverse Transcription Kit (Beyotime Biotechnology, Shanghai, China). Primer sequences for the housekeeping gene (*β-actin*) and target genes (*TYR* and *MITF*) are listed in Table 4. Relative quantification of *TYR* and *MITF* gene expression was analyzed using the 2^−ΔΔCt^ method.

### 4.9. Statistical Analysis

All experiments were performed with three independent replicates. Data are expressed as mean ± standard deviation. Statistical analyses were conducted using IBM SPSS Statistics 22.0 software (Armonk, NY, USA). Differences between groups were analyzed by one-way analysis of variance (ANOVA), with a significance level set at *p* < 0.05. Statistically significant differences among groups are indicated by different lowercase letters (e.g., a, b).

## 5. Conclusions

This study established an efficient two-stage enzymatic process for preparing glycopeptides CHAH from CHA, a by-product of liquid egg processing. The optimized protocol involved hydrolysis with 1.5% trypsin for 3 h, followed by 1% papain for 2 h. Under these conditions, CHAH was obtained with a high peptide yield and a sialic acid content of 1.96% (*w*/*w*). N-glycan profiling identified 14 distinct structures, most of which were sialylated or fucosylated. Both in vitro (B16 cells) and in vivo (zebrafish) models demonstrated that CHAH significantly inhibits tyrosinase activity and melanin synthesis in a concentration-dependent manner, without observable toxicity. Mechanistic studies revealed that these effects are mediated through the downregulation of key melanogenic genes, including MITF, TYR, TYRP1, and TYRP2. These findings elucidate the skin-whitening efficacy and molecular pathway of CHAH, providing a scientific foundation for its potential application in the skincare and functional food industries.

## Figures and Tables

**Table 1:** Putative Structures of N-Glycans in CHAH Glycopeptides.

Peak	N-Glycan Name	N-Glycan Structure
1	A3G3S3(2,6)	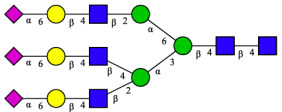
2	A2G2S2(2,6)	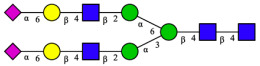
3	A2G2S1(2,6)S1(2,3)	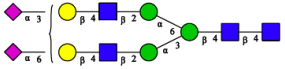
4	FA2G2S2(2,3)	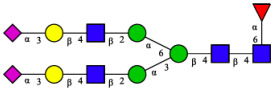
	A4G4S2(2,3)S1(2,6)	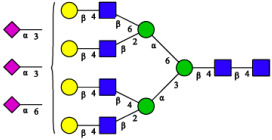
5	A2G1S1(2,6)[3]	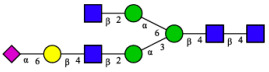
	A4F(3)G4S1(2,3)S2(2,6)	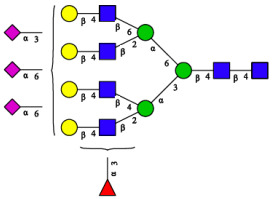
6	A3G3S1(2,3)S1(2,6)	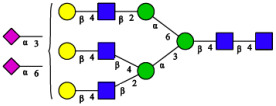
7	A2G2S1(2,6)	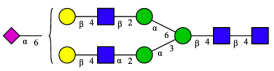
	FA2BG1S1(2,6)[3]	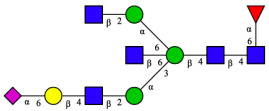
8	FA2G2S1(2,6)	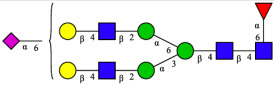
	Man5	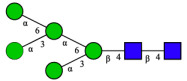
9	Man6	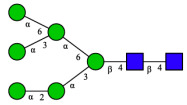
10	FA2G1[6]	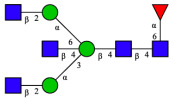
11	FA2G1[3]	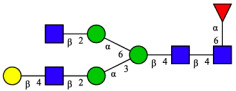
12	A2G2	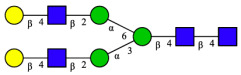
	FA2BG1[6]	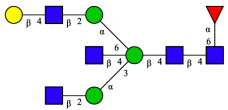
13	FA2BG2	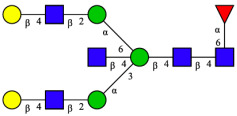
14	A3G3[2,4]	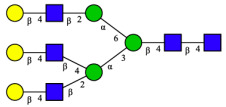

Note: The symbolic representation of N-glycan structures was generated using GlycoWorkbench version 1.1, adhering to the guidelines established by the Functional Glycomics Alliance [35].The names of glycans are based on the Oxford nomenclature. The table background has no special meaning; it is solely used to distinguish the structures represented by adjacent serial numbers, enabling clearer differentiation between them.

**Table 2:** Tyrosinase Inhibitor IC50 Comparison Chart.

Matter	IC50
CHAH	13.80 mg/mL
Edible Bird’s Nest Peptide	7.55 mg/mL
Arbutin	1.09 mM
L-Ascorbic Acid	12.5 μM
Kojic Acid	~2.8 μM

**Table 3:** Primer sequences of relevant genes in B16 melanoma cells.

Gene	Upstream Primers (5′-3′)	Downstream Primers (5′-3′)
*β-actin*	*GTGACGTTGACATCCGTAAAGA*	*GCCGGACTCATCGTACTCC*
*MITF*	*CCAACAGCCCTATGGCTATGC*	*CTGGGCACTCACTCTCTGC*
*TYR*	*CAAAGGGGTGGATGACCGTG*	*AACTTACAGTTTCCGCAGTTGA*
*TYRP1*	*CCCCTAGCCTATATCTCCCTTTT*	*TACCATCGTGGGGATAATGGC*
*TYRP2*	*CTTGGGGTTGCTGGCTTTTC*	*CGCTGAAGAGTTCCACCTGT*

Note: *MITF* refers to Microphthalmia-associated Transcription Factor; *TYR* refers to Tyrosinase; *TYRP1* refers to Tyrosinase-related protein 1; *TYRP2* refers to Tyrosinase-related protein 2.

**Table 4:** Oligonucleotide primers used for zebrafish genes in this study.

Gene	Upstream Primers (5′-3′)	Downstream Primers (5′-3′)
*β-actin*	*TCGAGCATGGAGATGGGAACC*	*CTCGTGGATACCGCAAGATTC*
*MITF*	*GGATACTTCATGGTGCCCTT*	*TCAGGAACTCCTGCACAAAC*
*TYR*	*CCTTCCCAGTCCTGACATGT*	*GTTCGTCCATACTGCTGCTG*

Note: *MITF* refers to Microphthalmia-associated Transcription Factor; *TYR* refers to Tyrosinase.

## Data Availability

The data presented in this study are available on request from the author.

## Supplementary Materials

The following supporting information can be downloaded at: https://www.mdpi.com/article/10.3390/molecules31010059/s1, Table S1: CHAH Molecular Weight Distribution.

## Abbreviations

The following abbreviations are used in this manuscript:

CHA	Chalaza
CHAH	CHA-derived glycopeptides
VC	L-ascorbic acid
PBS	Phosphate-buffered saline
DMSO	Dimethyl sulfoxide
FBS	Fetal bovine serum
DMEM	Dulbecco’s Modified Eagle Medium
ECM	Embryo culture medium
MWCO	Molecular weight cut-off
MITF	Microphthalmia-associated Transcription Factor
TYR	Tyrosinase
TYRP1	Tyrosinase-related protein 1
TYRP2	Tyrosinase-related protein 2
ROS	Reactive oxygen species
TNF-α	Tumor necrosis factor-α
IL-1β	Interleukin-1β
SOD	Superoxide dismutase
GSH-Px	Glutathione peroxidase
Neu5Ac	N-acetylneuraminic acid
OPD	o-phenylenediamine
HPLC	High-Performance Liquid Chromatography
DSA-FACE	DNA sequencer-assisted fluorophore-assisted carbohydrate electrophoresis
RT-PCR	Reverse transcription-polymerase chain reaction
CCK-8	Cell Counting Kit-8
DPPH	1,1-Diphenyl-2-picrylhydrazyl
ABTS	2,2′-Azinobis-(3-ethylbenzthiazoline-6-sulfonic acid)
ANOVA	One-way analysis of variance
DH	Degree of hydrolysis
hpf	Hours post-fertilization
TAA	Thioacetamide

## References

[1] Kawakami A., Fisher D.E. (2011). Key discoveries in melanocyte development. J. Investig. Dermatol..

[2] Hida T., Kamiya T., Kawakami A., Ogino J., Sohma H., Uhara H., Jimbow K. (2020). Elucidation of melanogenesis cascade for identifying pathophysiology and therapeutic approach of pigmentary disorders and melanoma. Int. J. Mol. Sci..

[3] Lynde C.B., Kraft J.N., Lynde C.W. (2006). Topical treatments for melasma and postinflammatory hyperpigmentation. Ski. Ther. Lett..

[4] Pillaiyar T., Manickam M., Jung S.-H. (2017). Downregulation of melanogenesis: Drug discovery and therapeutic options. Drug Discov. Today.

[5] Lee R., Ko H.J., Kim K., Sohn Y., Min S.Y., Kim J.A., Na D., Yeon J.H. (2020). Anti-melanogenic effects of extracellular vesicles derived from plant leaves and stems in mouse melanoma cells and human healthy skin. J. Extracell. Vesicles.

[6] Kobayashi T., Hearing V.J. (2007). Direct interaction of tyrosinase with Tyrp1 to form heterodimeric complexes in vivo. J. Cell Sci..

[7] Bai W., Deng F., Liu X., Yin X., Qiu X., Yang J., Yang W., Zhang X., Lian J., Fan Q. (2024). Edible bird’s nest peptide (EBNP) with high whitening activity: Sequences analysis, whitening activity characterization and molecular docking study. J. Funct. Foods.

[8] Liu F., Qu L., Li H., He J., Wang L., Fang Y., Yan X., Yang Q., Peng B., Wu W. (2022). Advances in biomedical functions of natural whitening substances in the treatment of skin pigmentation diseases. Pharmaceutics.

[9] Briganti S., Camera E., Picardo M. (2003). Chemical and instrumental approaches to treat hyperpigmentation. Pigment Cell Res..

[10] Stamford N.P.J. (2012). Stability, transdermal penetration, and cutaneous effects of ascorbic acid and its derivatives. J. Cosmet. Dermatol..

[11] Deng Y., Huang L., Zhang C., Xie P., Cheng J., Wang X., Liu L. (2020). Skin-care functions of peptides prepared from Chinese quince seed protein: Sequences analysis, tyrosinase inhibition and molecular docking study. Ind. Crops Prod..

[12] Qorbani A., Mubasher A., Sarantopoulos G.P., Nelson S., Fung M.A. (2020). Exogenous Ochronosis (EO): Skin lightening cream causing rare caviar-like lesion with banana-like pigments; review of literature and histological comparison with endogenous counterpart. Autops. Case Rep..

[13] Fujimoto N., Onodera H., Mitsumori K., Tamura T., Maruyama S., Ito A. (1999). Changes in thyroid function during development of thyroid hyperplasia induced by kojic acid in F344 rats. Carcinogenesis.

[14] Mishra P., Ahsan F., Mahmood T., Bano S., Ansari V.A., Yadav J., Ansari J.A., Khan M.M.U. (2025). Acute and Subacute Toxicity Study of α-Arbutin: An In Vivo Evidence. J. Appl. Toxicol..

[15] Song Y., Chen S., Li L., Zeng Y., Hu X. (2022). The hypopigmentation mechanism of tyrosinase inhibitory peptides derived from food proteins: An overview. Molecules.

[16] Gǎlbǎu C.-Ş., Irimie M., Neculau A.E., Dima L., Pogačnik da Silva L., Vârciu M., Badea M. (2024). The Potential of Plant Extracts Used in Cosmetic Product Applications—Antioxidants Delivery and Mechanism of Actions. Antioxidants.

[17] Bai W., Liu X., Fan Q., Lian J., Guo B. (2023). Study of the antiaging effects of bird’s nest peptide based on biochemical, cellular, and animal models. J. Funct. Foods.

[18] Huang P., Miao J., Liao W., Huang C., Chen B., Li Y., Wang X., Yu Y., Liang X., Zhao H. (2023). Rapid screening of novel tyrosinase inhibitory peptides from a pearl shell meat hydrolysate by molecular docking and the anti-melanin mechanism. Food Funct..

[19] Devita L., Lioe H.N., Nurilmala M., Suhartono M.T. (2021). The Bioactivity Prediction of Peptides from Tuna Skin Collagen Using Integrated Method Combining In Vitro and In Silico. Foods.

[20] Morakul B., Teeranachaideekul V., Wongrakpanich A., Leanpolchareanchai J. (2024). The evidence from in vitro primary fibroblasts and a randomized, double-blind, placebo-controlled clinical trial of tuna collagen peptides intake on skin health. J. Cosmet. Dermatol..

[21] Byun K.A., Lee S.Y., Oh S., Batsukh S., Jang J.W., Lee B.J., Rheu K.M., Li S., Jeong M.S., Son K.H. (2024). Fermented Fish Collagen Attenuates Melanogenesis via Decreasing UV-Induced Oxidative Stress. Mar. Drugs.

[22] Kose A., Oncel S.S. (2022). Design of melanogenesis regulatory peptides derived from phycocyanin of the microalgae Spirulina platensis. Peptides.

[23] Bodurlar Y., Caliskan M. (2022). Inhibitory activity of soybean (*Glycine max* L. Merr.) Cell Culture Extract on tyrosinase activity and melanin formation in alpha-melanocyte stimulating Hormone-Induced B16-F10 melanoma cells. Mol. Biol. Rep..

[24] Liu J., Wu Q., Yang T., Yang F., Guo T., Zhou Y., Han S., Luo Y., Guo T., Luo F. (2021). Bioactive peptide F2d isolated from rice residue exerts antioxidant effects via Nrf2 signaling pathway. Oxidative Med. Cell. Longev..

[25] Vichit W., Saewan N. (2022). Anti-Oxidant and Anti-Aging Activities of Callus Culture from Three Rice Varieties. Cosmetics.

[26] Fossa Shirata M.M., Maia Campos P.M.B.G. (2021). Sunscreens and cosmetic formulations containing ascorbyl tetraisopalmitate and rice peptides for the improvement of skin photoaging: A double-blind, randomized placebo-controlled clinical study. Photochem. Photobiol..

[27] Jia L., Wang L., Liu C., Liang Y., Lin Q. (2021). Bioactive peptides from foods: Production, function, and application. Food Funct..

[28] Rabl A., Spadaro J.V., Zoughaib A. (2008). Environmental impacts and costs of solid waste: A comparison of landfill and incineration. Waste Manag. Res..

[29] Pu J., Hu J., Xiao J., Li S., Wang B., Wang J., Geng F. (2024). Integrated landscape of chicken egg chalaza proteomics. Poult. Sci..

[30] Chan C.-J., Tseng J.-K., Wang S.-Y., Lin Y.-L., Wu Y.-H.S., Chen J.-W., Chen Y.-C. (2020). Ameliorative effects of functional chalaza hydrolysates prepared from protease-A digestion on cognitive dysfunction and brain oxidative damages. Poult. Sci..

[31] Lin Y.-L., Lu C.-F., Wu Y.-H.S., Yang K.-T., Yang W.-Y., Chen J.-W., Tseng J.-K., Chen Y.-C. (2021). Protective effects of crude chalaza hydrolysates against liver fibrogenesis via antioxidation, anti-inflammation/anti-fibrogenesis, and apoptosis promotion of damaged hepatocytes. Poult. Sci..

[32] Najafian L., Babji A. (2012). A review of fish-derived antioxidant and antimicrobial peptides: Their production, assessment, and applications. Peptides.

[33] Takada H., Katoh T., Katayama T. (2020). Sialylated O -Glycans from Hen Egg White Ovomucin are Decomposed by Mucin-degrading Gut Microbes. J. Appl. Glycosci..

[34] Laroy W., Contreras R., Callewaert N. (2006). Glycome mapping on DNA sequencing equipment. Nat. Protoc..

[35] Varki A., Cummings R.D., Esko J.D., Freeze H.H., Stanley P., Marth J.D., Bertozzi C.R., Hart G.W., Etzler M.E. (2009). Symbol nomenclature for glycan representation. Proteomics.

[36] Kim J.J., Kim K.S., Yu B.J. (2018). Optimization of antioxidant and skin-whitening compounds extraction condition from *Tenebrio molitor* larvae (mealworm). Molecules.

[37] Li X., Meng F., Sun T., Hao Z., Wang Y., Jiang Y., Wang Y., Li Y., Ding Y. (2025). Peptides from Dalian *Stichopus japonicus*: Antioxidant Activity and Melanogenesis Inhibition In Vitro Cell Models and In Vivo Zebrafish Models Guided by Molecular Docking Screening. Mar. Biotechnol..

[38] Pillaiyar T., Manickam M., Jung S.-H. (2017). Recent development of signaling pathways inhibitors of melanogenesis. Cell. Signal..

[39] Snyman M., Walsdorf R.E., Wix S.N., Gill J.G. (2024). The metabolism of melanin synthesis—From melanocytes to melanoma. Pigment Cell Melanoma Res..

[40] Cronin J.C., Wunderlich J., Loftus S.K., Prickett T.D., Wei X., Ridd K., Vemula S., Burrell A.S., Agrawal N.S., Lin J.C. (2009). Frequent mutations in the MITF pathway in melanoma. Pigment Cell Melanoma Res..

[41] Roider E., Lakatos A.I.T., McConnell A.M., Wang P., Mueller A., Kawakami A., Tsoi J., Szabolcs B.L., Ascsillán A.A., Suita Y. (2024). MITF regulates IDH1, NNT, and a transcriptional program protecting melanoma from reactive oxygen species. Sci. Rep..

[42] Wang G., Zhang J., Peng Z. (2025). Tyrosinase inhibitory mechanism of pyrimidine-thiols and their potential application in the anti-browning of fresh-cut apples. Food Chem..

[43] Ahuja K., Raju S., Dahiya S., Motiani R.K. (2025). ROS and calcium signaling are critical determinant of skin pigmentation. Cell Calcium.

[44] Lim D., Lee K.J., Kim Y., Kim M., Ju H.M., Kim M.J., Choi D.H., Choi J., Kim S., Kang D. (2021). A Basic Domain-Derived Tripeptide Inhibits MITF Activity by Reducing its Binding to the Promoter of Target Genes. J. Investig. Dermatol..

[45] Zhu L., Xiong H., Huang X., Guyonnet V., Ma M., Chen X., Zheng Y., Wang L., Hu G. (2022). Identification and molecular mechanisms of novel antioxidant peptides from two sources of eggshell membrane hydrolysates showing cytoprotection against oxidative stress: A combined in silico and in vitro study. Food Res. Int..

[46] Yubolphan R., Phongsiri K., Mongkhammee N., Pidech J., Roytrakul S., Suwattanasophon C., Choowongkomon K., Daduang S., Khunkitti W., Jangpromma N. (2025). Anti-Inflammatory Effects of Hen Egg White Hydrolysate and Its Specific Peptides IS8, PA11, and PK8 on LPS-Induced Macrophage Inflammation. Food Sci. Nutr..

[47] Yang K.T., Lin Y.L., Lin Y.X., Wang S.Y., Wu Y.H.S., Chou C.H., Fu S.G., Chen Y.C. (2018). Protective effects of antioxidant egg-chalaza hydrolysates against chronic alcohol consumption-induced liver steatosis in mice. J. Sci. Food Agric..

[48] Ak T., Gülçin I. (2008). Antioxidant and radical scavenging properties of curcumin. Chem.-Biol. Interact..

[49] You Y., Cao Y., Guo S., Xu J., Li Z., Wang J., Xue C. (2015). Purification and identification of α 2–3 linked sialoglycoprotein and α 2–6 linked sialoglycoprotein in edible bird’s nest. Eur. Food Res. Technol..

[50] Martín M.J., Vázquez E., Rueda R. (2007). Application of a sensitive fluorometric HPLC assay to determine the sialic acid content of infant formulas. Anal. Bioanal. Chem..

