# Supplementary material for: Preparation and Whitening Activity of Sialoglycopeptide of Chalaza from Liquid Egg Process
Source: Molecules. 2025 Dec 23;31(1):59. doi: 10.3390/molecules31010059 (PMC12786514; doi:10.3390/molecules31010059)
Supplement: Supplementary file 1 [file molecules-31-00059-s001.zip › molecules-4013757-supplementary.pdf]

Table S1: CHAH Molecular Weight Distribution

	migration time	Mn	Mw	MP	Peak Area	Peak Area Ratio (%)
**1**	12.262	1506008	1596783	1480844	613155	0.71
**2**	13.269	708983	742692	901237	211458	0.24
**3**	15.109	373550	374857	363852	15016	0.02
**4**	15.451	264999	271346	307421	36042	0.04
**5**	19.007	53069	53357	53224	808332	0.93
**6**	19.521	39034	39528	41317	701210	0.81
**7**	20.727	24253	24415	22795	439496	0.51
**8**	21.061	19405	19482	19337	452642	0.52
**9**	22.921	7308	7783	7726	12812618	14.81
**10**	23.817	4104	4176	4969	45927355	53.07
**11**	25.063	2722	2740	2687	4654352	5.38
**12**	25.593	2034	2049	2070	5265505	6.08
**13**	26.317	1424	1436	1449	9582339	11.07
**14**	27.102	975	982	983	2488654	2.88
**15**	27.977	588	616	639	2210360	2.55
**16**	29.911	251	253	246	140263	0.16
**17**	30.488	182	184	185	178927	0.21
